# Harnessing monocrop breeding strategies for intercrops

**DOI:** 10.3389/fpls.2024.1394413

**Published:** 2024-05-10

**Authors:** Reena Dubey, Riccardo Zustovi, Sofie Landschoot, Kevin Dewitte, Greet Verlinden, Geert Haesaert, Steven Maenhout

**Affiliations:** Department of Plants and Crops, Faculty of Bioscience Engineering, Ghent University, Ghent, Belgium

**Keywords:** plant breeding, intercrop, monocrop, compatibility traits, heritability, genetic gain

## Abstract

Intercropping is considered advantageous for many reasons, including increased yield stability, nutritional value and the provision of various regulating ecosystem services. However, intercropping also introduces diverse competition effects between the mixing partners, which can negatively impact their agronomic performance. Therefore, selecting complementary intercropping partners is the key to realizing a well-mixed crop production. Several specialized intercrop breeding concepts have been proposed to support the development of complementary varieties, but their practical implementation still needs to be improved. To lower this adoption threshold, we explore the potential of introducing minor adaptations to commonly used monocrop breeding strategies as an initial stepping stone towards implementing dedicated intercrop breeding schemes. While we acknowledge that recurrent selection for reciprocal mixing abilities is likely a more effective breeding paradigm to obtain genetic progress for intercrops, a well-considered adaptation of monoculture breeding strategies is far less intrusive concerning the design of the breeding programme and allows for balancing genetic gain for both monocrop and intercrop performance. The main idea is to develop compatible variety combinations by improving the monocrop performance in the two breeding pools in parallel and testing for intercrop performance in the later stages of selection. We show that the optimal stage for switching from monocrop to intercrop testing should be adapted to the specificity of the crop and the heritability of the traits involved. However, the genetic correlation between the monocrop and intercrop trait performance is the primary driver of the intercrop breeding scheme optimization process.

## 1 Introduction

Monoculture refers to the cultivation of only one crop species in a field at a time. This approach has been utilized extensively to maximize yields and streamline agricultural production for economically important sole crops (Power and Follett, 1987). It gained tremendous success in the sixties and aided in eradicating food shortages across the world. Since then, monocropping has evolved, giving birth to an intensive agriculture system that builds upon external inputs such as mineral fertilizers and crop protection products, improved varieties, mechanizations, and large-scale farming (Tilman, 2020). While monoculture offers numerous advantages, there is significant discussion regarding its long-term sustainability. It is argued that monoculture may neglect certain aspects of ecosystem dynamics and could potentially be less effective in meeting the present demands for resource efficiency (Altieri, 2002; Aggarwal, 2006; Brockerhoff et al., 2017; Forrester, 2017). This approach, while effective for maximizing short-term production, can have detrimental effects on the biological foundation of agricultural ecosystems. The continuous application of chemicals disrupts the delicate balance of soil microbial communities, beneficial insects and pest organisms, that are essential drivers of a sustainable agricultural system (Hole et al., 2005; Ge et al., 2008; Potts et al., 2010; Goulson et al., 2015; Hartmann et al., 2015; Sofo et al., 2020). Such disruptions can compromise soil fertility, reduce biodiversity, and contaminate critical biological assets in farming landscapes, raising concerns about the long-term viability and ecological impact of these practices (Sánchez-Bayo, 2011; Mandal et al., 2020).

An increasing number of agronomists are advocating for a reassessment of cultivation techniques and a shift towards agricultural practices that are more ecologically sustainable (Coolman and Hoyt, 1993; Brooker et al., 2015; Hathaway, 2016; Dang et al., 2020; Leclère et al., 2020). One such recommended approach is intercropping. This technique involves the cultivation of two or more crops near one another in the same field, with the goal of enhancing yield and other desirable traits while maintaining a balanced ecosystem (Brooker et al., 2015; Gaba et al., 2015; Weih et al., 2022b). It encompasses a variety of methods based on the cropping pattern of different species, representing within field diversity. Types of intercropping include various practices such as mixed intercropping, where different crops are grown simultaneously without distinct row patterns; row intercropping, which involves planting different crops in separate rows; strip intercropping, where crops are cultivated in distinct strips; and relay intercropping, which features different crops grown during overlapping periods (Andrews and Kassam, 1976; Mousavi and Eskandari, 2011; Neamatollahi et al., 2013).

Previous studies demonstrate that mixed crops frequently yield higher in low nitrogen fertilization conditions and more consistent returns per unit area, improve diets and produce greater returns than pure stands particularly when legumes are involved (Cordero and McCollum, 1976; Francis and Sanders, 1978; Willey, 1979; Francis, 1981; Beets, 1982; Pearce and Edmondson, 1984; Li et al., 2023; Landschoot et al., 2024; Zustovi et al., 2024). The results vary when nitrogen fertilization is introduced as the availability of nitrogen influences both the productivity and the cooperative interactions between cereal and legume crops (Jensen, 1996; Ghanbari-Bonjar and Lee, 2002; Andersen et al., 2005; Hauggaard-Nielsen and Jensen, 2005; Kebede, 2021; Weih et al., 2021). While an increase in nitrogen usually leads to higher biomass production, it often reduces the complementarity between the crops, as the cereal tends to dominate and suppress the legume component (Ofori and Stern, 1987; Midmore, 1993; Andersen et al., 2005; Ghaley et al., 2005; Iannetta et al., 2016; Raseduzzaman and Jensen, 2017; Kebede, 2021).

The seed market currently offers limited options for authentic seed mixes designed for intercropping, particularly for cereal and legume combinations where the delicate interplay between supply and demand can be identified as the main limiting factor. Most of the available mixes cater to forage crops and turfs, being marketed as legume-grass, cover crop and polyculture lawn mixes. The development of commercial mixes for cereal-legume intercropping faces challenges, primarily due to the lack of established formulations and the difficulty in accurately describing the performance of various varieties under intercropping conditions. This complexity arises from the vast potential for creating different mix compositions. Research demonstrates that intercropping higher-yielding cultivars that are developed for monocrop systems do not always produce optimal results (Hamblin and de Oliveira Zimmermann, 1986; Hill, 1990; O’Leary and Smith, 1999; Brooker et al., 2015; Annicchiarico et al., 2019).

However, developing dedicated intercrop varieties requires specialized breeding strategies that are generally incompatible with monocrop breeding objectives (Wright, 1985; Hill, 1997; Li et al., 2014; Brooker et al., 2015; Litrico and Violle, 2015; Sampoux et al., 2020; Annicchiarico et al., 2021; Bourke et al., 2021; Ergon and Bakken, 2022; Moore et al., 2022; Weih et al., 2022a). Intercropping selection schemes introduce significant breeding challenges due to the large number of pairwise combinations of selection candidates from the two breeding pools and their complex interspecific interactions that are further compounded by environmental factors such as soil type, weather patterns and pest and disease pressure (Acquaah, 2009).

Traits that are critical to successful intercropping, like root architecture and flowering time, are both genetically complex and sensitive to environmental influences, yet require meticulous finetuning and synchronization between the intercrop partners. Environmental interaction further complicates the evaluation of intercrops as multiple trial environments are required to identify combinations that consistently yield well together. It is generally accepted that addressing these challenges requires specialized breeding strategies that involve extensive crossing schemes and dedicated evaluation strategies to identify superior-performing variety combinations (Francis and Smith, 1985; Wright, 1985; Davis and Woolley, 1993; Nelson and Robichaux, 1997; Moore et al., 2022). These dedicated intercrop breeding strategies are inherently more time-consuming and resource-intensive than conventional monocrop breeding schemes. The required overhaul of the breeding scheme likely explains why intercrop breeding remains an academic matter with, to our knowledge, no practical implementations in commercial breeding companies.

This study aims to introduce minimal changes to well-known monocrop breeding schemes as a stepping stone towards more dedicated intercrop breeding approaches. Regardless of the chosen breeding scheme, selecting for intercrop performance assumes that in one or more selection stages, the combined phenotypic response of the intercrop will be assessed. Introducing intercrop phenotyping early in the selection process allows to maximize the complementarity of the mixing partners but reduces the number of candidates that can be tested due to the quadratic number of pairwise variety combinations that can be formed in binary intercrops. Postponing intercrop testing to the later stages increases the initial size of the two sets of selection candidates, but it implies an initial selection on performance in monocropping conditions, which only partially reflects their intercrop capabilities. The primary objective of this study is to pinpoint the optimal stage of a conventional, monocrop breeding program at which one can switch to intercrop selection, assuming fixed phenotyping resources at every stage. We examine scenarios with varying levels of heritability and different genetic correlations between monocrop and intercrop performance. Recommendations for the optimal stage in which to select for intercrop traits will be provided.

## 2 Materials and methods

Using a stochastic simulation framework, two different monocrop breeding schemes are adapted for intercrop breeding. We assume a cereal-legume intercropping system with hypothetical crop components similar to faba bean (*Vicia faba*) and triticale (*x Triticosecale* Wittm.). Both these species are partially allogamous but in our simulations we breed them as self-pollinating crops for the creation of fixed lines as is common practice. All breeding programs are simulated using the AlphasimR package (Gaynor et al., 2021) using version 4.2.2 of the R software (R Core Team, 2020). Computations were performed on a server equipped with 96 cores, with each simulation run comprising 100 independent replications for each scenario (**Supplementary Material 2.1**). The total run time for all simulations was approximately 27 hours.

### 2.1 Genome simulation

Haplotype sequences for both faba bean and triticale founder populations were simulated using Markovian Coalescent Simulator (MaCS) implemented in AlphaSimR, recreating the evolutionary process with multiple cycles of drift, mutation and selection (Chen et al., 2009). Notably, while our simulations were specifically conducted with faba bean and triticale, it’s important to emphasize that the framework we developed is adaptable and applicable to other crops as well. In total, 100 homozygous individuals of each crop were simulated to form the founder population. For computational efficiency, the genome of both component crops, triticale (an allo-polyploid) and faba bean (a diploid), has been simulated as a single diploid chromosome. For triticale, the physical length of the single chromosome was assumed to be 9.9e+08 basepair (bp), which is the average physical size of triticale’s wheat (Avni et al., 2017) and rye (Li et al., 2021) chromosomes. For the faba bean, the physical length of the single chromosome was assumed to be 1.1e+9 bp (Jayakodi et al., 2023).

### 2.2 Trait simulation

In each scenario and for the two component crops, two correlated traits were simulated, representing the monocrop (MC) and intercrop (IC) performance of the breeding pool accessions. These traits were assumed to adhere to an additive genetic model that is controlled by 1000 biallelic QTL at polymorphic sites that are randomly distributed over the single chromosome and for which a reference allele effect is sampled from the standard normal distribution (Gaynor et al., 2021).

Genetic values (GVs) for both the MC and IC trait are computed through the summation of the QTL effects across all segregating sites. The loci influencing MC and IC trait performance are assumed to be identical but the different allele dosage effects are sampled in a way that fixes the genetic correlation between the two traits to one of three values from the set {0.3, 0.5, 0.9}. It should be clear that a positive correlation is required to allow for indirect selection for IC performance through evaluation of the MC response. The GV of an IC combination is obtained by averaging the IC GV of the faba bean and the triticale component as shown in Equation 1.


(1) $$\begin{array}{c} g=\frac{g_{f\;}+g_{t}}{2}\;, \end{array}$$

Where, *g* represents the GV of the IC combination, *g_f_* denotes the IC GV of the faba bean component and *g_t_* refers to the IC GV of the triticale component.

Phenotypes were simulated by adding a random environmental effect to the GVs of the MC and IC traits. These random environmental effects have been sampled from a normal distribution with zero mean and a variance $\frac{\sigma_{e}^{2}}{r}$ that matches the heritability of the trait and the number of replications *r* at a particular selection stage following Equation 2.


(2) $$\begin{array}{c} \sigma_{e}^{2}=\frac{\left(1-h^{2}\right)\sigma_{a}^{2}}{h^{2}}\; \end{array}$$

Where, *h* ^2^ is the narrow sense heritability, $\sigma_{a}^{2}$ the additive genetic variance of the founder population, $\sigma_{e}^{2}$ the environmental variance.

### 2.3 MC breeding schemes

This study focuses on two distinct MC breeding strategies for self-pollinating crops used as pure lines:

• **Doubled Haploid** (DH) breeding accelerates variety development by producing homozygous inbred lines in one or two generations, reducing the need for several cycles of selfing or backcrossing (Thomas et al., 2003; Dwivedi et al., 2015; Humphreys and Knox, 2015). DHs exhibit complete genetic uniformity, enabling the capture and stabilization of desirable traits more efficiently (Yan et al., 2017). Its use in controlled crosses enables the precise combination of desirable traits, developing high-performing varieties (Pratap et al., 2010, 2018). Techniques for generating DH have been applied to nearly 400 species so far, resulting in the global introduction of more than 300 DH-based varieties across 12 different species (Seguí-Simarro et al., 2021; Weyen, 2021). With its speed, efficiency, genetic stability, and trait evaluation benefits, DH breeding can be a favoured method in commercial IC breeding programs, facilitating the timely delivery of improved varieties to meet the needs of farmers and consumers.
• **Ear/pod-to-row** (EPR) breeding is an adaptation of the classic ear-row breeding method, a variation of pedigree breeding technique used in plant breeding in which selection is carried out in progenies derived from individual ears. This method was initially developed by Hopkins for corn and further modified by Lonnquist and Compton and Comstock (Hopkins, 1899; Lonnquist, 1964; Compton and Comstock, 1976). Nowadays ear to row method is routinely used in wheat, rice, barley among other crops (Yamamoto, 1972; Lammerts van Bueren et al., 2011; Greveniotis et al., 2019; Weissmann et al., 2023). In our adapted approach, we select ears/pods from single plants with desirable traits and plant their seeds in separate rows in the subsequent growing season. Seeds from these ear/pods undergo phenotypic evaluation and selection for IC trait, superior individuals within the families are selected, increasing the frequency of favorable alleles and enhancing trait performance.

### 2.4 Breeding plan/experimental design

**Figure 1:**
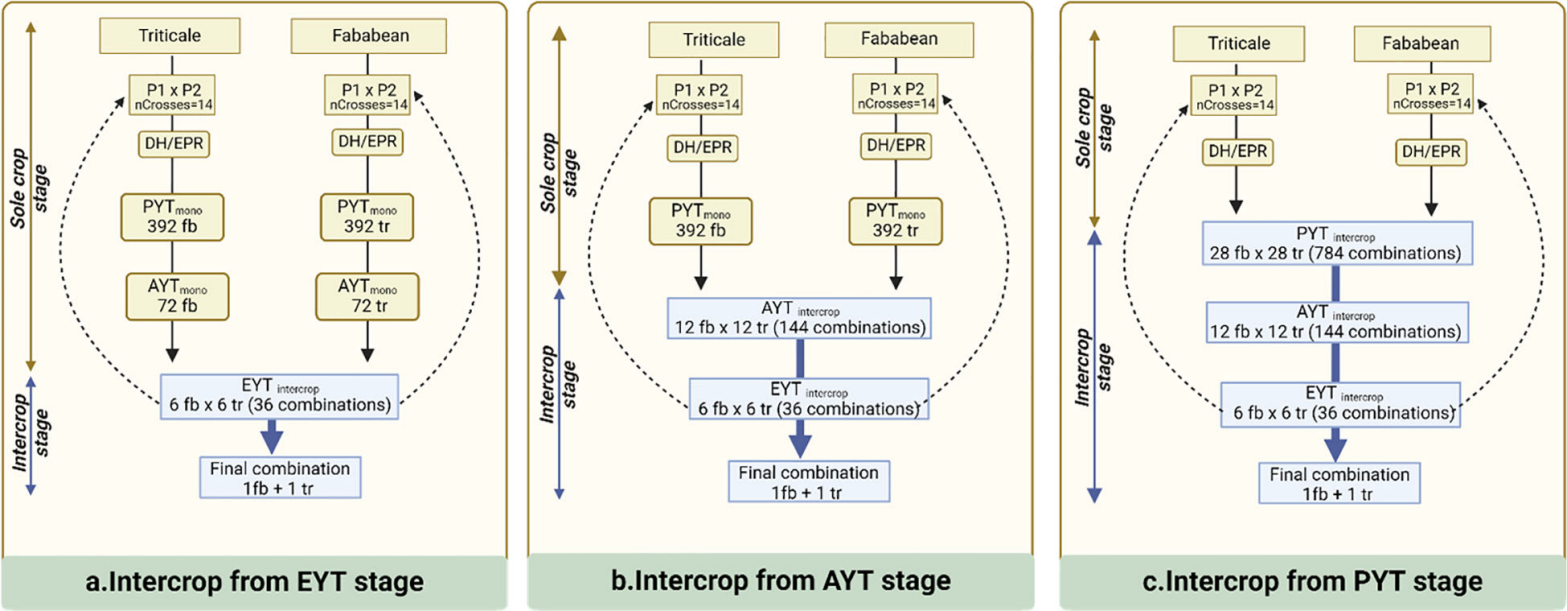
Outline of selection methods for intercrop breeding. **(A)** Intercrop trial and selection begins at preliminary yield trial stage (PYT) with total 784 combinations being tested. **(B)** Intercrop trial and selection begins at advanced yield trial stage (AYT) with total 144 combinations being tested. **(C)** Intercrop trial and selection begins at elite yield trial stage (EYT) with total 36 combinations being tested. Successive parents for the next cycles are sampled from EYT stage.

**Figure 1** provides a schematic overview of the various breeding schemes that are examined in this study and have been partitioned according to the selection stage at which IC testing is introduced. In this breeding program, we assume there are three key selection stages: a preliminary yield trial (PYT), an advanced yield trial (AYT) and an elite yield trial (EYT). The PYT represents the initial screening stage, where a large number of lines are phenotypically evaluated without replication. AYT is the second stage, where promising lines from PYT are tested in two replications. Finally, the EYT represents the last phase, during which the top-performing lines from the AYT are assessed for potential commercial release. The presented breeding schemes have been specifically designed to have identical resource requirements for phenotyping in each of the selection stages. This implies that all schemes are constrained in the same way with respect to the number of candidate accessions that can be tested, enabling a fair comparison in terms of selection efficiency. Breeding schemes do differ in the number of initial crosses and the number of selected candidates at the end of each stage. The initial phase involves the creation of 14 initial crosses for both faba bean (Fb) and triticale (Tr). To facilitate these crosses, 14 parents are randomly sampled from the founder population of 100 individuals for each crop. In the EPR-based selection schemes, the 14 S0 plants are consecutively selfed until S4 assuming a visual, within family selection with low heritability (*h^2^ = *0.1). Subsequently, 8 percent of the individuals, 28 in total, are advanced to the preliminary yield trials. In the DH-based breeding schemes, the S0 individuals are assumed to produce fully homozygous inbred lines (DH0) without selection. In the MC scenario, 392 Fb and 392 Tr S4/DH0 plants enter PYT hosting a total of 784 single replicate plots on a single location. In the scenario where IC testing is introduced at PYT, 28 fb lines are combined with 28 tr lines, giving rise to 784 unique Fb x Tr line combinations.

Following the PYT, AYT allows to test 144 entries in two replicates, involving either 12 Fb x 12 Tr line combinations in the IC scenario or 72 Fb and 72 Tr lines in the MC setting. In the following EYT, there is room to test 36 IC combinations from 6 Fb and 6 Tr lines in four replicates. The lines that make it to the AYT stage are recycled as parents for making new initial crosses in the subsequent breeding cycle. In each scenario, a total of 20 breeding cycles are simulated to monitor genetic progress, derived from the IC GVs during EYT stage, involving 36 IC combinations.

## 3 Results

### 3.1 Genetic progress

**Figure 2:**
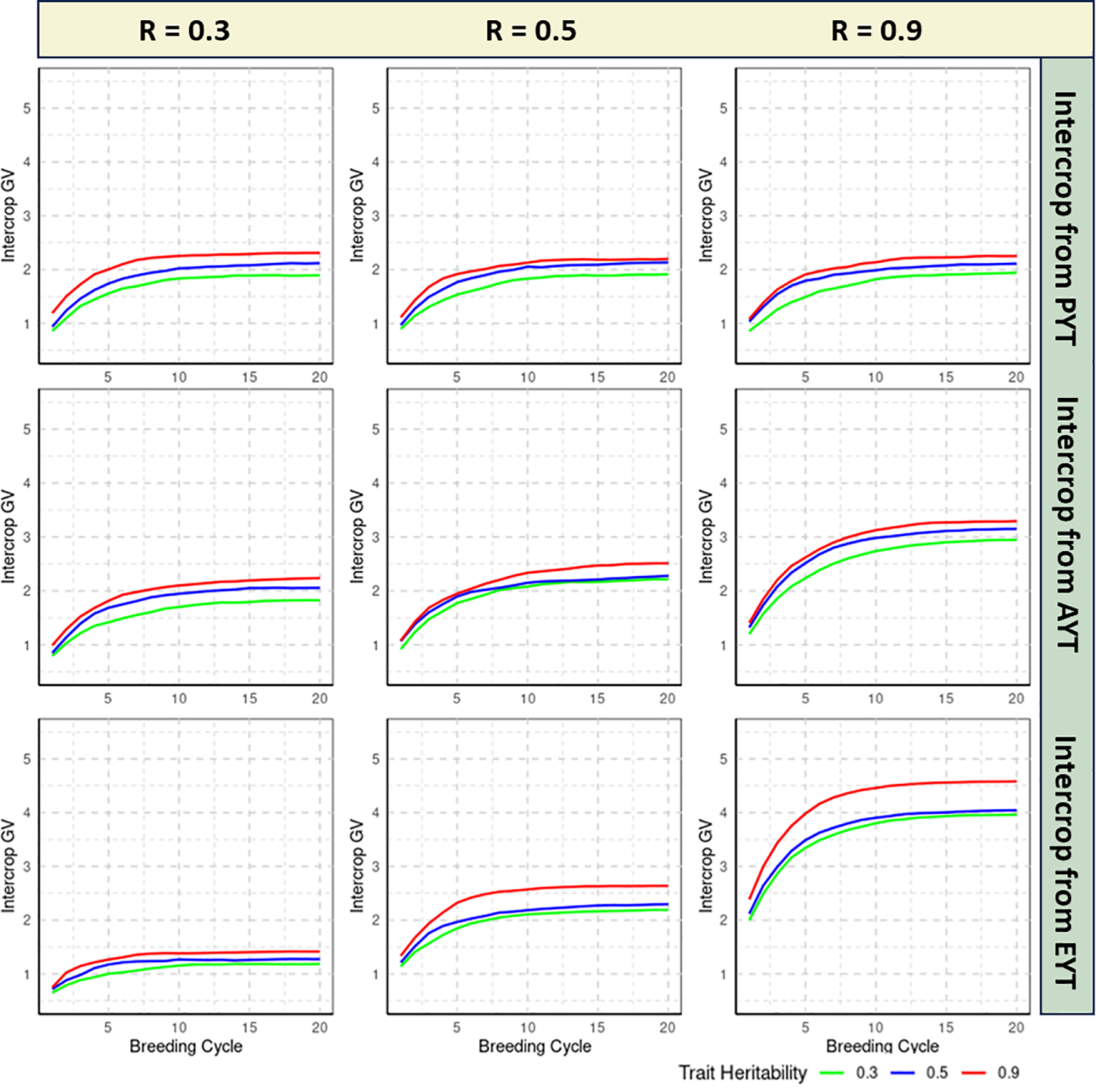
Genetic progress realized by the double haploid (DH) breeding scheme as measured by the intercrop (IC) genetic values (GVs) over 20 breeding cycles, shown for three levels of trait heritability (0.3, 0.5, 0.9) and different genetic correlations between MC and IC trait performance (0.3, 0.5, 0.9). Phenotypic evaluation of IC performance is either introduced in the Preliminary Yield Trial (PYT), the Advanced Yield Trial (AYT) or the Elite Yield Trial (EYT) selection stages. The realized genetic progress for each breeding method is quantified by plotting the mean IC GVs of the 36 IC combinations at the EYT stage, averaged over 100 independent iterations of the simulation routine.

**Figure 3:**
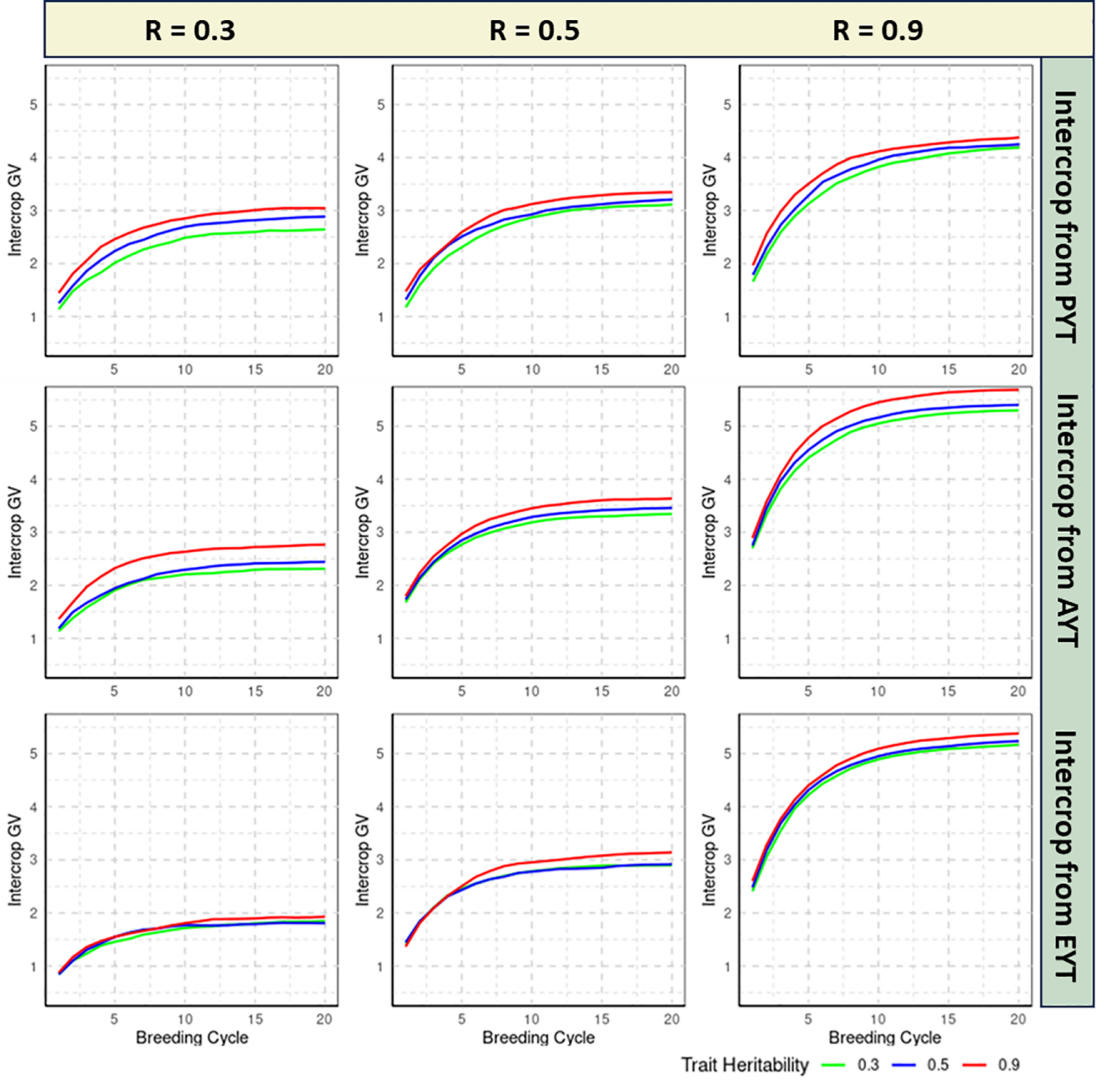
Genetic progress realized by ear/pod-to-row (EPR) breeding scheme as measured by the intercrop (IC) genetic values (GVs) over 20 breeding cycles, shown for three levels of trait heritability (0.3, 0.5, 0.9) and different genetic correlations between MC and IC trait performance (0.3, 0.5, 0.9). Phenotypic evaluation of IC performance is either introduced in the Preliminary Yield Trial (PYT), the Advanced Yield Trial (AYT) or the Elite Yield Trial (EYT) selection stages. The realized genetic progress for each breeding method is quantified by plotting the mean IC GVs of the 36 IC combinations at the EYT stage, averaged over 100 independent iterations of the simulation routine.

The realized genetic progress of each breeding scheme is quantified by calculating the average IC GVs of the 36 IC combinations that are tested at the EYT stage for each of the 20 consecutive breeding cycles. All reported estimates are averaged over 100 independent iterations of the simulation routine. **Figures 2**, **3** specifies the IC GVs for three levels of trait heritability (0.3, 0.5, 0.9) and three levels of genetic correlation between MC and IC trait performance (R = 0.3, R = 0.5, R = 0.9). Phenotypic evaluation of IC performance is either introduced in the PYT, AYT or EYT selection stages, as described in **Figure 1**.

In all simulated scenarios, GVs increase over the initial breeding cycles and converge asymptotically to a maximum value. Convergence is assumed when the difference between the average GVs of the IC trait between two consecutive breeding cycles becomes smaller than 0.005 (**Supplementary Material Table 1**).

Genetic variances in IC trait diminishes consistently across all scenarios, showing a gradual decline as breeding cycles advances (**Supplementary Material 2.2**).

This asymptotic behavior of GV is readily explained by the gradual depletion of genetic variance over breeding cycles, as these schemes do not implement a variance preserving strategy like those advocated by Allier et al., 2020 and Vanavermaete et al., 2021. For this particular study, however, both the initial rate of genetic progress and the asymptotic limit are informative criteria to compare the efficiency of the examined selection schemes under different assumptions of trait heritability and genetic correlation between MC and IC trait performance.

#### 3.1.1 DH breeding method

If the genetic correlation between MC and IC performance is low (i.e. R = 0.3), genetic progress of IC GVs is slow, regardless of the heritability of the trait. In this setting, postponing IC phenotyping until the final EYT stage is detrimental for the maximum reachable GV for all three examined heritability levels (**Figure 2**). In this scenario, IC testing should be introduced in the earliest PYT stage albeit the difference with an introduction at AYT stage is small, especially for the highly heritable trait. Convergence to the maximum reachable GV typically occurs between 8-19 breeding cycles in different scenarios of DH breeding (**Supplementary Material Table 1**).

If the genetic correlation between MC and IC performance is moderate (i.e. R = 0.5), introducing IC testing in the PYT stage is least efficient in terms of the maximum GV that is reached and the number of breeding cycles that are required to converge to this maximum value, regardless of the heritability of the trait under study. When IC testing is delayed until the AYT or EYT stages, the pattern of genetic progress is quite similar for low and medium heritability traits. For the highly heritable trait, however, the maximum GV is higher for the EYT scenario compared to the AYT scenario. The latter reaches its maximum value after 13,16,14 breeding cycles for PYT, AYT and EYT selection stages (**Supplementary Material Table 1**). This implies that for moderate genetic correlation scenarios, an introduction of IC phenotyping in the EYT stage can be designated as the preferred strategy due to higher GVs, regardless of the heritability of the trait.

This conclusion is even more pronounced when the genetic correlation between MC and IC performance is high (i.e. R = 0.9), where the asymptotic GV is higher and converge faster when IC testing is introduced at the EYT stage, irrespective of the trait heritability. Convergence of GVs in this scenario for highly correlated traits can be observed between 12-19 breeding cycles for all traits irrespective of heritabilities.

#### 3.1.2 EPR breeding approach

The examination of genetic correlations across different heritability scenarios reveals multifaceted insights into how the genetic architecture and selection effectiveness interact within the EPR breeding method. If the genetic correlation between MC and IC performance is low (i.e. R = 0.3), genetic progress of IC GVs is slow, regardless of the heritability of the trait. Convergence to the maximum reachable GV typically occurs between 12- 20 breeding cycles in different scenarios of EPR breeding(**Supplementary Material Table 1**). In this setting, postponing IC phenotyping until the final EYT stage is detrimental for the maximum reachable value for all three examined heritability levels. IC testing should be introduced in the earliest PYT stage but the difference with an introduction at AYT stage is small, especially for highly heritable traits (**Figure 3**).

The scenario that assumes a moderate genetic correlation (i.e. R = 0.5) between MC and IC trait performance reveals that the maximum reachable GV is realized when IC testing is introduced at the AYT stage, for all the trait heritability. Convergence of GV starts at the 19th breeding cycle for PYT selection, and at the 16th breeding cycle for AYT and EYT selection(**Supplementary Material Table 1**). Contrary to the DH breeding method, postponing IC testing to the EYT stage decreases the maximum IC GVs for all trait heritabilities.

If the genetic correlation between MC and IC performance is strong, meaning R=0.9, we see a pattern similar to that of R=0.5. In this scenario, the highest GV for IC is achieved at the AYT stage across all levels of heritability. However, this advantage is more significant for traits with high heritability. For traits with low to medium heritability, phenotyping for IC can commence either at the AYT or the EYT stage. In this scenario, selection based on PYT converges after 20,16,19 breeding cycles respectively for low medium and high correlated traits, whereas for the AYT and EYT selection stages, the maximum achievable GV starts converging between 16 to 19 breeding cycles(**Supplementary Material Table 1**).

#### 3.1.3 Comparison between DH and EPR breeding

DH breeding resulted in lower maximum GVs compared to EPR breeding scheme in all scenarios, implying that DH is a less efficient breeding strategy. This comparison is, however, biased in favor of the EPR strategy as this scheme encompasses four cycles of within-family visual selection during fixation of the selection candidates that enter the PYT stage In the DH breeding scheme, the DH0 candidates directly enter the PYT stage without any form of preselection. This disadvantage of the DH breeding approach cannot be compensated for as both schemes are assumed to have identical phenotyping resources from the PYT stage onwards.

In the DH breeding schemes, regardless of the stage at which IC testing is introduced, GVs approach their asymptotic maximum starting after approximately 8 breeding cycles for R =0.3 and 12 for R= 0.5 and 0.9. In the EPR breeding schemes, introduction of IC selection in the AYT and EYT stages results in GVs that approach their maximum value starting at 13, 15 and 16 breeding cycles for R=0.3, R = 0.5 and R = 0.9 respectively.

The impact of the genetic correlation between MC and IC performance on the realized selection response differs markedly between the two breeding methods. In EPR breeding, the higher genetic correlation (R = 0.9) results in a substantial increase in the maximum reachable GV, implying a strong selection response. Conversely, in the DH method, the effect of the genetic correlation is less pronounced, which is likely also related to the lack of an explicit preselection stage which constrains the maximum reachable GV.

## 4 Discussion

Research on IC breeding methodology has revived in recent years, as demonstrated by several publications (Lithourgidis et al., 2011; Duc et al., 2015; Bančič et al., 2021; Moore et al., 2022) that build upon the foundations that were laid out in the seventies and eighties of the previous century (Hamblin et al., 1976; Francis, 1981; Wright, 1985). Two main breeding concepts have gradually crystallized over this era. The producer-associate concept (Haug et al., 2021, 2023) involves the producer effect, representing a genotype’s impact on its yield, and the associate effect, reflecting its influence on the companion crop. This approach allows for a detailed analysis of competitive abilities within mixtures, emphasizing the importance of both individual yield components and IC compatibilities. This concept enables breeders to optimize mixtures for specific ratios, shaping overall performance through IC trait optimization.

A more practical approach to tackling the complex of ICs involves the selection of candidates based on their reciprocal mixing abilities (Wright, 1985). Inspired by the concept of combining abilities in hybrid breeding programs, a mixing ability represents the average IC performance of an accession when combined with multiple accessions of the complementary crop. This approach, initially used for studying plant interactions, has been widely applied to predict the agronomic performance of binary mixtures. The methodology has been developed and refined over several decades, with significant contributions from researchers such as (Sprague and Tatum, 1942; Griffing, 1956; Jensen and Federer, 1965), and others throughout the 1960s and beyond, highlighting its broad applicability and refinement over time (McGilchrist, 1965; Chalbi, 1967; Gallais, 1970; Federer, 1979; Federer et al., 1982; Gizlice et al., 1989; Knott and Mundt, 1990; Gallandt et al., 2001; Forst et al., 2019; Haug et al., 2021). Reciprocal mixing ability refers to the compatibility between different crop species or varieties to support each other’s growth when planted together. In each cycle, joint selection occurs for both species, considering the performance of pairs of progeny families from selected candidates. At the beginning of each breeding cycle, the candidates from selected IC pairs of the previous cycle are recombined within each species to form the selection population for the next cycle (Sampoux et al., 2020). By selecting varieties with high reciprocal mixing ability, farmers can maximize the advantages of intercropping, such as increased land productivity and enhanced nutrient utilization (Barot et al., 2017; Fan et al., 2020). For instance, the selection of a tall cereal crop alongside a shorter legume utilizes the vertical space efficiently and reduces the ground area needed for cultivation. In return, the legume can fix atmospheric nitrogen which also benefits the cereal. In addition, the selected component crop also benefits from the diverse nutrient requirements and rooting depths. This complementary relationship maximizes land use and can lead to higher total yields than if the crops were grown separately (Vandermeer, 1989; Altieri et al., 2018).

Breeding strategies for IC should prioritize enhancing crop performance within multi-species systems, emphasizing the importance of complementarity and synergy. The effectiveness of IC systems is typically evaluated using performance metrics such as the Land Equivalent Ratio, instead of solely focusing on the yield of individual crop species (Bedoussac and Justes, 2011; Zustovi et al., 2024). Although there is ongoing debate among researchers regarding the development of appropriate IC breeding methods, there remains a lack of evidence of their practical feasibility (Bourke et al., 2021; Moore et al., 2022; Weih et al., 2022a, Weih et al., 2022b; Moore et al., 2023). While there have been a few studies aimed at breeding legumes for intercropping, such as those by (Annicchiarico et al., 2021) and (Ergon and Bakken, 2022), these methods do not address the simultaneous improvement of both crops involved in the intercropping system. In silico studies by Bančič et al. (2021) demonstrate that genomic selection could significantly accelerate genetic gains in IC breeding (1.3-2.5 times) compared to phenotypic selection, with a notable influence of the genetic correlations (0.4, 0.7, 0.9). The presented Grid-Genomic selection excelled at low correlations, advocating a combined monocrop-intercrop approach for improved selection accuracy. The proposed method could benefit the breeders but the simplified assumptions in the studies might not reflect the complex interactions that IC breeders face. In our study, we similarly advocate a combined monocrop and intercrop approach but emphasize the ease of its adoption by breeders.

Despite these advancements, the IC breeding process remains challenging, requiring a complete overhaul of the MC breeding scheme, generally implying a considerable increase of phenotypic or genomic evaluation efforts. It is likely that the current market demand for intercrop varieties does not provide sufficient economic incentive to establish these dedicated intercrop breeding programs. This observation suggests that minor adaptations of existing MC breeding strategies could offer a more practical pathway towards IC breeding, potentially yielding faster results while minimizing the challenges and risks. While the selection efficiency of dedicated IC breeding strategies (Wright, 1985; Bančič et al., 2021; Moore et al., 2023) is likely superior to that of the proposed MC adaptations, the latter could represent the low hanging fruit with respect to the realization of commercial IC varieties.

### 4.1 Optimizing MC breeding for practical IC breeding

IC breeding presents challenges due to the complicated interactions and involvement of various species (Thierfelder et al., 2012; Brooker et al., 2015; Bourke et al., 2021; Huss et al., 2022; Weih et al., 2022b). To facilitate this process, a simplified and customizable breeding strategy is required, one that can be adapted for a wide range of crops and is straightforward to implement. Numerous publications underscore the critical need for an adaptable breeding strategy to enable effective IC breeding (Annicchiarico et al., 2019; Bourke et al., 2021; Weih et al., 2022a; Moore et al., 2023). Our research aimed to determine the most suitable selection stage for transitioning from MC breeding to IC breeding. All examined breeding schemes assume phenotypic selection with a fixed number of available field plots at each selection stage, maintaining a constant phenotyping workload during both MC and IC settings. The presented approach facilitates the integration of IC trait selection into MC breeding programs without significant increases in resource allocation, suggesting a methodologically sound and economically viable pathway for enhancing crop yields and sustainability through IC breeding.

### 4.2 Trait correlations between MC and IC genetic values impact genetic progress

The relationship between MC and IC performance, specifically in terms of trait correlations, has been a consistent focus, with research over the decades affirming a existence of variable correlation among crops in MC and IC setting (Atwood and Garber, 1942; Dijkstra and De Vos, 1972; Gomez and Gomez, 1983; Caradus et al., 1989; de Oliveira Zimmermann, 1997; Holland and Brummer, 1999; Santalla et al., 2001; Gebeyehu et al., 2006; Maamouri et al., 2017; Homulle et al., 2021; MacLaren et al., 2023). Gomez and Gomez (1983) reviewed 30 studies comparing yields of MC and IC systems for cereals, legumes, and sweet potatoes. For cereals, correlations in yield variations across 28 varieties ranged from 0.35 to 0.90. Legume trials with 19 varieties, showed a wider correlation range from -0.36 to 0.91, these findings underline the critical role of trait correlations in IC performance. In this study, the influence of the genetic correlations between MC and IC performance is assessed in DH and EPR breeding schemes for a range of trait heritabilities, replicating real-world IC traits.

In scenarios characterized by a high genetic correlation (R = 0.9), our findings consistently advocate for the initiation of IC phenotyping at the EYT stage for traits spanning the full heritability range. This conclusion is readily explained by the observation that selection for MC performance in the PYT and AYT stages allows to test a much larger set of candidates while the high genetic correlation between MC and IC traits guarantees an indirect selection for IC performance. Furthermore, delaying IC phenotyping efforts until the EYT stage implies a considerable simplification of the phenotypic evaluation activities, making it easier to integrate IC breeding into existing MC programs. Results from previous studies also suggest that the calculation of a correlation coefficient between MC and IC can give an indication whether separate breeding efforts are necessary, if correlation is high and significant, there probably is no need for a separate breeding effort for IC improvement (Gomez and Gomez, 1983; Francis and Smith, 1985).

For traits that show a moderate genetic correlation (R = 0.5), our results indicate that IC phenotyping should be introduced at the AYT stage or later for traits of low to medium heritability. The findings of previous experiments suggest that there exists a moderate correlation between the MC performance of plant cultivars and their performance when grown in IC environment. This observation was consistently noted across different studies, particularly when pure stand performances were documented (Atwood and Garber, 1942; Caradus et al., 1989). Conversely, for traits with high heritability, IC phenotyping should be initiated at the EYT stage to maximize genetic progress. High heritability implies that the variation in these traits is largely due to genetic differences rather than environmental factors. A strong genetic signal makes it possible to delay intensive IC phenotyping efforts until the EYT stage, where the focus shifts to refining and selecting the top-performing lines. By this stage, breeding programs have already filtered out lines based on preliminary assessments, allowing resources to be concentrated on the most promising IC combinations. Low and medium heritability traits require more IC phenotyping efforts to identify the superior combinations.

The results of this study illustrate that for traits characterized by a low genetic correlation (R = 0.3), it is imperative to initiate IC phenotyping at the PYT stage, irrespective of trait heritabilities. or breeding method (i.e. DH or EPR). Given the low genetic correlation between MC and IC genotype performance, it becomes clear that selecting for MC performance does not allow to drive improvement in the IC trait. This is particularly challenging for breeding programs because genotypes that perform well in MC conditions might not exhibit the same level of performance or adaptability in intercropping setups. This discrepancy necessitates early initiation of IC phenotyping.

In this situation, postponing IC phenotyping until the EYT stage limits the opportunity to accurately select candidate genotypes for traits that are beneficial in intercropping systems early in the breeding process. Since genetic progress for IC traits is slow due to the low correlation with MC traits, waiting until the later stages of breeding to focus on IC performance risks overlooking lines that could have shown promising IC traits earlier. This delay can hinder the ability to achieve the maximum genetic potential for these traits, affecting the overall efficiency and effectiveness of breeding program.

In our study, we considered specific correlation level between MC and IC traits, however, it is essential to recognize that real world scenarios introduce additional complexities. For instance, a study conducted in Colombia examined the performance of climbing bean cultivars across two consecutive seasons, revealing a high correlation between yields in both systems during the first season (R = 0.82), while the correlation was lower in the subsequent season (R = 0.41). Similarly, yields of bush bean cultivars exhibited a high correlation in the initial season (R = 0.88) and a lower correlation in the following season (R = 0.51). Additionally, mungbean data from the International Rice Research Institute (IRRI) in the Philippines depicted similar results (Francis and Sanders, 1978; Francis and Smith, 1985).

Intercrop breeding for perennials faces unique challenges, notably due to their longer lifecycles and complex root systems, demanding innovative but patient strategies for effective selection and optimization. A recent review by Moore et al., 2022 highlights distinct objectives across various intercropping systems, focusing on optimizing productivity within specific forage mixtures, breeders aim to enhance the overall productivity of grass and legume mixes harvested together. Perennial grain-forage systems prioritize grain yield, with forage as a secondary aim. Strategies vary across systems, emphasizing temporal and spatial niche differentiation to reduce competition and enhance complementarity. These findings underscore the dynamic nature of intercropping systems and the importance of considering variability across seasons and cultivars in optimizing IC breeding schemes.

### 4.3 Transition to dedicated IC breeding

The suggested adaptations to existing MC breeding schemes should be considered as a first, low-threshold step towards dedicated IC breeding strategies. The next step likely involves the use of tester varieties to reduce the number of IC combinations that are evaluated in the field. In this setup, all selection candidates of one component crop are intercropped with a single accession of the other component crop. In hybrid breeding programs, this single accession is referred to as the tester line which is specifically chosen for its superior combining ability when crossed with accessions of the complementary heterotic group. This scheme translates to a selection for general mixing ability in an IC setting which is expected to increase IC selection efficiency.

### 4.4 Limitations of stochastic simulations

While discussing the implications of our findings, it is critical to acknowledge the characteristic limitations of stochastic simulations. While these routines are instrumental for the theoretical optimization of breeding schemes, they inevitably simplify the complicated genetic interactions and environmental variables that characterize real-world scenarios.

Our simulations, predicated on various assumptions, may not encapsulate the entire spectrum of gene-environment interaction effects, particularly in the dynamic setting of intercropping. Although phenotype-based selection is employed as a realistic stand-in for assessing genetic potential, real-life phenotypes can be influenced by genotype-by-environment interactions which have not been considered in this study. Furthermore, most breeders are confronted with multiple-trait objectives that cover a range of heritabilities and as such obfuscate the optimal stage for intercrop (IC) testing. However, the commonly used multi-trait selection indexes allow to aggregate multiple trait objectives in a univariate pseudo-trait that is generally cursed with a low heritability but nonetheless adheres to the breeder’s equation and is therefore covered by the presented simulation scenarios. A similar logic can be followed for other commonly used multi-trait selection strategies such as independent culling or tandem selection that also represent specific aggregations on the involved traits.

It should also be noted that the cost and time per breeding cycle can differ substantially between selection strategies such as EPR and DH. This study, however, assumes that both approaches incur identical costs prior to field testing. However, the cost associated with DH breeding largely depends on the specificities of the applied DH protocol and the cost of EPR is a function of the number of selfing generations and the applied protocol for phenotypic evaluation prior to field testing. A more in-depth comparison of the EPR and DH selection strategies would require detailed cost estimates of the involved pre-testing steps for the crop combination under study.

Real-world breeding programs are also shaped by a confluence of factors beyond genetic data, including ecological considerations, economic viability and the decision-makers’ expertise, which are beyond the scope of our simulation models. Consequently, while our simulation-based approach yields valuable insights for prospective IC breeders, these insights must be corroborated through empirical research to ensure their practical relevance and accuracy in actual breeding programs.

## 5 Conclusion

In the presented study, we employed triticale and faba bean as model organisms within a cereal-legume intercropping system. Our analytical simulations uniquely focused on one property—the average physical size of the chromosomes of these crops. This implies that the presented framework is easily adapted to other species combinations, although we acknowledge that variations in chromosome size among component crops could influence the outcomes differently. Our results demonstrate that minor yet strategic modifications to MC breeding schemes allow to breed for IC performance through extensive stochastic simulations, we pinpoint the most suitable stage for switching from MC to IC phenotyping. These results can be used to guide the transition from MC breeding to IC breeding, maximizing the realised rate of IC genetic progress within the scope of a practically feasible breeding scheme. This foundational work is instrumental in guiding future IC breeding strategies, facilitating a strategic and genetically optimized transition from MC to IC breeding, thereby contributing significantly to the advancement of sustainable agricultural practices.

## Data availability statement

The original contributions presented in the study are available on GitHub, repository link: https://github.com/UGENT-Predictive-breeding/rdubey.

## Author contributions

RD: Conceptualization, Formal analysis, Investigation, Methodology, Writing – original draft, Writing – review & editing. RZ: Writing – review & editing. SL: Writing – review & editing. KD: Writing – review & editing. GV: Writing – review & editing. GH: Funding acquisition, Methodology, Supervision, Writing – review & editing. SM: Conceptualization, Methodology, Resources, Supervision, Validation, Writing – review & editing.

## References

[B1] Acquaah G. (2009). Principles of plant genetics and breeding (Hoboken, New Jersey, USA: John Wiley & Sons).

[B2] Aggarwal R. M. (2006). Globalization, local ecosystems, and the rural poor. World Dev. 34, 1405–1418. doi: 10.1016/j.worlddev.2005.10.011

[B3] Allier A. Teyssèdre S. Lehermeier C. Moreau L. Charcosset A. (2020). Optimized breeding strategies to harness genetic resources with different performance levels. BMC Genomics 21, 1–16. doi: 10.1186/s12864-020-6756-0 PMC7216646 32393177

[B4] Altieri M. A. (2002). Agroecology: the science of natural resource management for poor farmers in marginal environments. Agriculture Ecosyst. Environ. 93, 1–24. doi: 10.1016/S0167-8809(02)00085-3

[B5] Altieri M. A. Farrell J. G. Hecht S. B. Liebman M. Magdoff F. Murphy B.. (2018). “The agroecosystem: determinants, resources, processes, and sustainability,” in Agroecology (Boca Raton, FL, United States: CRC Press), 41–68. doi: 10.1201/9780429495465

[B6] Andersen M. K. Hauggaard-Nielsen H. Ambus P. Jensen E. S. (2005). Biomass production, symbiotic nitrogen fixation and inorganic N use in dual and tri-component annual intercrops. Plant Soil 266, 273–287. doi: 10.1007/s11104-005-0997-1

[B7] Andrews D. Kassam A. (1976). The importance of multiple cropping in increasing world food supplies. Multiple Cropping 27, 1–10. doi: 10.2134/asaspecpub27.c1

[B8] Annicchiarico P. Collins R. P. De Ron A. M. Firmat C. Litrico I. Hauggaard-Nielsen H. (2019). Do we need specific breeding for legume-based mixtures? Adv. Agron. 157, 141–215. doi: 10.1016/bs.agron.2019.04.001

[B9] Annicchiarico P. Nazzicari N. Notario T. Martin C. M. Romani M. Ferrari B.. (2021). Pea breeding for intercropping with cereals: variation for competitive ability and associated traits, and assessment of phenotypic and genomic selection strategies. Front. Plant Sci. 12, 731949. doi: 10.3389/fpls.2021.731949 34630481 PMC8495324

[B10] Atwood S. Garber R. (1942). The evaluation of individual plants of white clover for yielding ability in association with bluegrass. Agron. J. 34 (1), 1–6. doi: 10.2134/agronj1942.00021962003400010001x

[B11] Avni R. Nave M. Barad O. Baruch K. Twardziok S. O. Gundlach H.. (2017). Wild emmer genome architecture and diversity elucidate wheat evolution and domestication. Science 357, 93–97. doi: 10.1126/science.aan0032 28684525

[B12] Bančič J. Werner C. R. Gaynor R. C. Gorjanc G. Odeny D. A. Ojulong H. F.. (2021). Modeling illustrates that genomic selection provides new opportunities for intercrop breeding. Front. Plant Sci. 12, 605172. doi: 10.3389/fpls.2021.605172 33633761 PMC7902002

[B13] Barot S. Allard V. Cantarel A. Enjalbert J. Gauffreteau A. Goldringer I.. (2017). Designing mixtures of varieties for multifunctional agriculture with the help of ecology. A review. Agron. Sustain. Dev. 37 (2), 13. doi: 10.1007/s13593-017-0418-x

[B14] Bedoussac L. Justes E. (2011). A comparison of commonly used indices for evaluating species interactions and intercrop efficiency: Application to durum wheat–winter pea intercrops. Field Crops Res. 124, 25–36. doi: 10.1016/j.fcr.2011.05.025

[B15] Beets W. C. (1982). Multiple cropping and tropical farming system, grower (Colorado, USA: London, Britain, and West views press). *156p*.

[B16] Bourke P. M. Evers J. B. Bijma P. Apeldoorn van D. F. Smulders M. J. M. Kuyper T. W.. (2021). Breeding beyond monoculture: putting the “intercrop” into crops. Front. Plant Sci. 12, 734167. doi: 10.3389/fpls.2021.734167 34868116 PMC8636715

[B17] Brockerhoff E. G. Barbaro L. Castagneyrol B. Forrester D. I. Gardiner B. González-Olabarria J. R.. (2017). Forest biodiversity, ecosystem functioning and the provision of ecosystem services. Biodiversity Conserv. 26, 3005–3035. doi: 10.1007/s10531-017-1453-2

[B18] Brooker R. W. Bennett A. E. Cong W.-F. Daniell T. J. George T. S. Hallett P. D.. (2015). Improving intercropping: a synthesis of research in agronomy, plant physiology and ecology. New Phytol. 206, 107–117. doi: 10.1111/nph.13132 25866856

[B19] Caradus J. R. Mackay A. C. Van Den Bosch J. Woodfield D. R. (1989). Comparative evaluation of white clover cultivars in spaced plant and small mixed species plot trials. New Z. J. Agric. Res. 32, 433–436. doi: 10.1080/00288233.1989.10421763

[B20] Chalbi N. (1967). ‘La compétition entre génotypes et ses effets sur les caractères quantitatifs de la Luzerne’. Ann. Amélior. Plantes 17, 67–82.

[B21] Chen G. K. Marjoram P. Wall J. D. (2009). Fast and flexible simulation of DNA sequence data. Genome Res. 19, 136–142. doi: 10.1101/gr.083634.108 19029539 PMC2612967

[B22] Compton W. A. Comstock R. E. (1976). More on modified ear-to-row selection in corn1. Crop Sci. 16, 122–122. doi: 10.2135/cropsci1976.0011183X001600010034x

[B23] Coolman R. M. Hoyt G. D. (1993). Increasing sustainability by intercropping. HortTechnology 3, 309–312. doi: 10.21273/HORTTECH.3.3.309

[B24] Cordero A. McCollum R. E. (1976). Intercropping research in North Carolina. Annual report. Agronomic-economic research on soils in the tropics 77, 181–200.

[B25] Dang K. Gong X. Zhao G. Wang H. Ivanistau A. Feng B. (2020). Intercropping alters the soil microbial diversity and community to facilitate nitrogen assimilation: a potential mechanism for increasing proso millet grain yield. Front. Microbiol. 11, 601054. doi: 10.3389/fmicb.2020.601054 33324383 PMC7721675

[B26] Davis J. H. C. Woolley J. N. (1993). Genotypic requirement for intercropping. Field Crops Res. 34, 407–430. doi: 10.1016/0378-4290(93)90124-6

[B27] de Oliveira Zimmermann M. J. (1997). “Breeding for yield, in mixtures of common beans (Phaseolus vulgaris L.) and maize (Zea mays L.),” in Adaptation in Plant Breeding: Selected Papers from the XIV EUCARPIA Congress on Adaptation in Plant Breeding held at Jyväskylä, Sweden from July 31 to August 4, 1995 (Dordrecht: Springer), 143–148.

[B28] Dijkstra J. De Vos A. (1972). The evaluation of selections of white clover (Trifolium repens L.) inmonoculture and in mixture with grass. Euphytica 21, 432–449. doi: 10.1007/BF00039339

[B29] Duc G. Agrama H. Bao S. Berger J. Bourion V. De Ron A. M.. (2015). Breeding annual grain legumes for sustainable agriculture: new methods to approach complex traits and target new cultivar ideotypes. Crit. Rev. Plant Sci. 34, 381–411. doi: 10.1080/07352689.2014.898469

[B30] Dwivedi S. L. Britt A. B. Tripathi L. Sharma S. Upadhyaya H. D. Ortiz R. (2015). Haploids: constraints and opportunities in plant breeding. Biotechnol. Adv. 33, 812–829. doi: 10.1016/j.biotechadv.2015.07.001 26165969

[B31] Ergon Å. Bakken A. K. (2022). Breeding for intercropping: the case of red clover persistence in grasslands. Euphytica 218, 98. doi: 10.1007/s10681-022-03051-7

[B32] Fan Y. Wang Z. Liao D. Raza M. A. Wang B. Zhang J.. (2020). Uptake and utilization of nitrogen, phosphorus and potassium as related to yield advantage in maize-soybean intercropping under different row configurations. Sci. Rep. 10, 9504. doi: 10.1038/s41598-020-66459-y 32528144 PMC7290029

[B33] Federer W. (1979). Statistical designs and response models for mixtures of cultivars 1. Agron. J. 71, 701–706. doi: 10.2134/agronj1979.00021962007100050003x

[B34] Federer W. T. Connigale J. C. Rutger J. N. Wijesinha A. (1982). Statistical analyses of yields from uniblends and biblends of eight dry bean cultivars. Crop Sci. 22, 111–115. doi: 10.2135/cropsci1982.0011183X002200010026x

[B35] Forrester D. I. (2017). Ecological and physiological processes in mixed versus monospecific stands. Mixed-species forests: Ecol. Manage., 73–115. doi: 10.1007/978-3-662-54553-9_3

[B36] Forst E. Enjalbert J. Allard V. Ambroise C. Krissaane I. Mary-Huard T. (2019). A generalized statistical framework to assess mixing ability from incomplete mixing designs using binary or higher order variety mixtures and application to wheat. Field Crops Res. 242, 107571. doi: 10.1016/j.fcr.2019.107571

[B37] Francis C. A. (1981). Development of plant genotypes for multiple cropping systems. Plant Breed. II, 179–232.

[B38] Francis C. A. Sanders J. H. (1978). Economic analysis of bean and maize systems: monoculture versus associated cropping. Field Crops Res. 1, 319–335. doi: 10.1016/0378-4290(78)90034-5

[B39] Francis C. A. Smith M. E. (1985). Variety development for multiple cropping systems. Crit. Rev. Plant Sci. 3, 133–168. doi: 10.1080/07352688509382207

[B40] Gaba S. Lescourret F. Boudsocq S. Enjalbert J. Hinsinger P. Journet E.-P.. (2015). Multiple cropping systems as drivers for providing multiple ecosystem services: from concepts to design. Agron. Sustain. Dev. 35, 607–623. doi: 10.1007/s13593-014-0272-z

[B41] Gallais A. (1970). Modèle pour l’analyse des relations d’associations binaires. Biométrie-Praximétrie 11, 51–80.

[B42] Gallandt E. R. Dofing S. M. Reisenauer E. P. Donaldson E. (2001). Diallel analysis of cultivar mixtures in winter wheat. Crop Sci. 41, 792–796. doi: 10.2135/cropsci2001.413792x

[B43] Gaynor R. C. Gorjanc G. Hickey J. M. (2021). AlphaSimR: an R package for breeding program simulations. G3 11, jkaa017. doi: 10.1093/g3journal/jkaa017 33704430 PMC8022926

[B44] Ge Y. Zhang J.-b. Zhang L.-m. Yang M. He J.-z. (2008). Long-term fertilization regimes affect bacterial community structure and diversity of an agricultural soil in northern China. J. Soils Sediments 8, 43–50. doi: 10.1065/jss2008.01.270

[B45] Gebeyehu S. Simane B. Kirkby R. (2006). Genotype× cropping system interaction in climbing beans (Phaseolus vulgaris L.) grown as sole crop and in association with maize (Zea mays L.). Eur. J. Agron. 24, 396–403. doi: 10.1016/j.eja.2006.01.005

[B46] Ghaley B. B. Hauggaard-Nielsen H. Høgh-Jensen H. Jensen Steen E. (2005). Intercropping of wheat and pea as influenced by nitrogen fertilization. Nutrient Cycling Agroecosystems 73, 201–212. doi: 10.1007/s10705-005-2475-9

[B47] Ghanbari-Bonjar A. Lee H. (2002). Intercropped field beans (Vicia faba) and wheat (Triticum aestivum) for whole crop forage: effect of nitrogen on forage yield and quality. J. Agric. Sci. 138, 311–315. doi: 10.1017/S0021859602002149

[B48] Gizlice Z. Carter T. E. Jr. Burton J. W. Emigh T. H. (1989). Partitioning of blending ability using two-way blends and component lines of soybean. Crop Sci. 29, 885–889. doi: 10.2135/cropsci1989.0011183X002900040008x

[B49] Gomez A. A. Gomez K. A. (1983). Multiple cropping in the humid tropics of Asia (Ottawa, Canada: International development research centre).

[B50] Goulson D. Nicholls E. Botías C. Rotheray E. L. (2015). Bee declines driven by combined stress from parasites, pesticides, and lack of flowers. Science 347, 1255957. doi: 10.1126/science.1255957 25721506

[B51] Greveniotis V. Zotis S. Sioki E. Ipsilandis C. G. (2019). Improving pedigree selection in applied breeding of barley populations. Cereal Res. Commun. 47, 123–133. doi: 10.1556/0806.46.2018.068

[B52] Griffing B. (1956). Concept of general and specific combining ability in relation to diallel crossing systems. Aust. J. Biol. Sci. 9, 463–493. doi: 10.1071/BI9560463

[B53] Hamblin J. Rowell J. G. Redden R. (1976). Selection for mixed cropping. Euphytica 25, 97–106. doi: 10.1007/BF00041533

[B54] Hamblin J. de Oliveira Zimmermann M. J. (1986). “Breeding common bean for yield in mixtures,” in Plant Breeding Reviews, Volume 4, Wiley Online Books, (INC Westport, Connecticut: Avi Publishing company), 245–272.

[B55] Hartmann M. Frey B. Mayer J. Mäder P. Widmer F. (2015). Distinct soil microbial diversity under long-term organic and conventional farming. ISME J. 9, 1177–1194. doi: 10.1038/ismej.2014.210 25350160 PMC4409162

[B56] Hathaway M. D. (2016). Agroecology and permaculture: addressing key ecological problems by rethinking and redesigning agricultural systems. J. Environ. Stud. Sci. 6, 239–250. doi: 10.1007/s13412-015-0254-8

[B57] Haug B. Messmer M. M. Enjalbert J. Goldringer I. Flutre T. Mary-Huard T.. (2021). Advances in breeding for mixed cropping–incomplete factorials and the producer/associate concept. Front. Plant Sci. 11, 620400. doi: 10.3389/fpls.2020.620400 33505418 PMC7829252

[B58] Haug B. Messmer M. M. Enjalbert J. Goldringer I. Flutre T. Mary-Huard T. (2023). New insights towards breeding for mixed cropping of spring pea and barley to increase yield and yield stability. Field Crops Res. 297, 108923. doi: 10.1016/j.fcr.2023.108923

[B59] Hauggaard-Nielsen H. Jensen E. S. (2005). Facilitative root interactions in intercrops. Root physiology: From Gene to Funct. 4, 237–250. doi: 10.1007/1-4020-4099-7_13

[B60] Hill J. (1990). The three C’s—competition, coexistence and coevolution—and their impact on the breeding of forage crop mixtures. Theor. Appl. Genet. 79, 168–176. doi: 10.1007/BF00225947 24226214

[B61] Hill J. (1997). “Breeding components for mixture performance,” in Adaptation in Plant Breeding: Selected Papers from the XIV EUCARPIA Congress on Adaptation in Plant Breeding held at Jyväskylä, Sweden from July 31 to August 4, 1995 (Jyväskylä, Sweden: Springer), 149–152.

[B62] Hole D. G. Perkins A. J. Wilson J. D. Alexander I. H. Grice P. V. Evans A. D. (2005). Does organic farming benefit biodiversity? Biol. Conserv. 122, 113–130. doi: 10.1016/j.biocon.2004.07.018

[B63] Holland J. Brummer E. (1999). Cultivar effects on oat–berseem clover intercrops. Agron. J. 91, 321–329. doi: 10.2134/agronj1999.00021962009100020023x

[B64] Homulle Z. George T. S. Karley A. J. (2021). Root traits with team benefits: understanding belowground interactions in intercropping systems. Plant Soil, 1–26. doi: 10.1007/s11104-021-05165-8 34720209

[B65] Hopkins C. G. (1899). Improvement in the chemical composition of the corn kernel. J. Am. Chem. Soc. 21, 1039–1057. doi: 10.1021/ja02061a012

[B66] Humphreys D. G. Knox R. E. (2015). Doubled haploid breeding in cereals. Adv. Plant Breed. strategies: breeding Biotechnol. Mol. Tools, 241–290. doi: 10.1007/978-3-319-22521-0_9

[B67] Huss C. Holmes K. Blubaugh C. (2022). Benefits and risks of intercropping for crop resilience and pest management. J. Economic Entomology 115, 1350–1362. doi: 10.1093/jee/toac045 35452091

[B68] Iannetta P. P. M. Young M. Bachinger J. Bergkvist G. Doltra J. Lopez-Bellido R. J.. (2016). A comparative nitrogen balance and productivity analysis of legume and non-legume supported cropping systems: the potential role of biological nitrogen fixation. Front. Plant Sci. 7. doi: 10.3389/fpls.2016.01700 PMC5116563 27917178

[B69] Jayakodi M. Golicz A. A. Kreplak J. Fechete L. I. Angra D. Bednář P.. (2023). The giant diploid faba genome unlocks variation in a global protein crop. Nature 615, 652–659. doi: 10.1038/s41586-023-05791-5 36890232 PMC10033403

[B70] Jensen E. (1996). Barley uptake of N deposited in the rhizosphere of associated field pea. Soil Biol. Biochem. 28, 159–168. doi: 10.1016/0038-0717(95)00134-4

[B71] Jensen N. Federer W. (1965). Competing ability in wheat 1. Crop Sci. 5, 449–452. doi: 10.2135/cropsci1965.0011183X000500050022x

[B72] Kebede E. (2021). Contribution, utilization, and improvement of legumes-driven biological nitrogen fixation in agricultural systems. Front. Sustain. Food Syst. 5. doi: 10.3389/fsufs.2021.767998

[B73] Knott E. Mundt C. (1990). Mixing ability analysis of wheat cultivar mixtures under diseased and nondiseased conditions. Theor. Appl. Genet. 80, 313–320. doi: 10.1007/BF00210065 24220962

[B74] Lammerts van Bueren E. T. Jones S. S Tamm L. Murphy K. M. Myers J. R. Leifert C.. (2011). The need to breed crop varieties suitable for organic farming, using wheat, tomato and broccoli as examples: A review. NJAS: Wageningen J. Life Sci. 58, 193–205. doi: 10.1016/j.njas.2010.04.001

[B75] Landschoot S. Zustovi R. Dewitte K. Randall N. P. Maenhout S. Haesaert G. (2024). Cereal-legume intercropping: a smart review using topic modelling. Front. Plant Sci. 14, 1228850. doi: 10.3389/fpls.2023.1228850 38259927 PMC10800527

[B76] Leclère D. Obersteiner M. Barrett M. Butchart S. H. M. Chaudhary A. De Palma A.. (2020). Bending the curve of terrestrial biodiversity needs an integrated strategy. Nature 585, 551–556. doi: 10.1038/s41586-020-2705-y 32908312

[B77] Li L. Tilman D. Lambers H. Zhang F.-S. (2014). Plant diversity and overyielding: insights from belowground facilitation of intercropping in agriculture. New Phytol. 203, 63–69. doi: 10.1111/nph.12778 25013876

[B78] Li G. Wang L. Yang J. He H. Jin H. Li X.. (2021). A high-quality genome assembly highlights rye genomic characteristics and agronomically important genes. Nat. Genet. 53, 574–584. doi: 10.1038/s41588-021-00808-z 33737755 PMC8035075

[B79] Li C. Stomph T.-J. Makowski D. Li H. Zhang C. Zhang F.. (2023). The productive performance of intercropping. Proc. Natl. Acad. Sci. 120, e2201886120. doi: 10.1073/pnas.2201886120 36595678 PMC9926256

[B80] Lithourgidis A. S. Dordas C. A. Damalas C. A. Vlachostergios D. N. (2011). Annual intercrops: an alternative pathway for sustainable agriculture. Aust. J. Crop Sci. 5, 396–410. doi: 10.3316/informit.281409060336481

[B81] Litrico I. Violle C. (2015). Diversity in plant breeding: a new conceptual framework. Trends Plant Sci. 20, 604–613. doi: 10.1016/j.tplants.2015.07.007 26440430

[B82] Lonnquist J. H. (1964). A modification of the ear-to-row procedure for the improvement of maize populations1. Crop Sci. 4, 227–228. doi: 10.2135/cropsci1964.0011183X000400020033x

[B83] Maamouri A. Louarn G. Béguier V. Julier B. (2017). Performance of lucerne genotypes for biomass production and nitrogen content differs in monoculture and in mixture with grasses and is partly predicted from traits recorded on isolated plants. Crop Pasture Sci. 68, 942–951. doi: 10.1071/CP17052

[B84] MacLaren C. Waswa W. Aliyu K. T. Claessens L. Mead A. Schöb C.. (2023). Predicting intercrop competition, facilitation, and productivity from simple functional traits. Field Crops Res. 297, 108926. doi: 10.1016/j.fcr.2023.108926

[B85] Mandal A. Sarkar B. Mandal S. Vithanage M. Patra A. K. Manna M. C. (2020). “Impact of agrochemicals on soil health,” in Agrochemicals detection, treatment and remediation (Oxford, United Kingdom: Elsevier), 161–187. doi: 10.1016/B978-0-08-103017-2.00007-6

[B86] McGilchrist C. (1965). Analysis of competition experiments. Biometrics 21, 975–985. doi: 10.2307/2528258

[B87] Midmore D. J. (1993). Agronomic modification of resource use and intercrop productivity. Field Crops Res. 34, 357–380. doi: 10.1016/0378-4290(93)90122-4

[B88] Moore V. M. Schlautman B. Fei S.-z. Roberts L. M. Wolfe M. Ryan M. R.. (2022). Plant breeding for intercropping in temperate field crop systems: a review. Front. Plant Sci. 13, 843065. doi: 10.3389/fpls.2022.843065 35432391 PMC9009171

[B89] Moore V. M.. (2023). Toward plant breeding for multicrop systems. Proc. Natl. Acad. Sci. 120, e2205792119. doi: 10.1073/pnas.2205792119 36972435 PMC10083599

[B90] Mousavi S. R. Eskandari H. (2011). A general overview on intercropping and its advantages in sustainable agriculture. J. Appl. Environ. Biol. Sci. 1, 482–486. Available at: https://api.semanticscholar.org/CorpusID:49522032.

[B91] Neamatollahi E. Jahansuz M. R. Mazaheri D. Bannayan M. (2013). “Intercropping,” in Sustainable agriculture reviews: volume 12 (Dordrecht: Springer Science+Business Media), 119–142.

[B92] Nelson S. Robichaux R. (1997). Identifying plant architectural traits associated with yield under intercropping: Implications of genotype-cropping system interactions. Plant Breed. 116, 163–170. doi: 10.1111/j.1439-0523.1997.tb02172.x

[B93] Ofori F. Stern W. R. (1987). Cereal–legume intercropping systems. Adv. Agron. 41, 41–90. doi: 10.1016/S0065-2113(08)60802-0

[B94] O’Leary N. Smith M. E. (1999). Breeding corn for adaptation to two diverse intercropping companions. Am. J. Altern. Agric. 14, 158–164. doi: 10.1017/S0889189300008328

[B95] Pearce S. C. Edmondson R. N. (1984). Experimenting with intercrops. Biometrics 40, 231–237. doi: 10.2307/2530764

[B96] Potts S. G. Biesmeijer J. C. Kremen C. Neumann P. Schweiger O. Kunin W. E. (2010). Global pollinator declines: trends, impacts and drivers. Trends Ecol. Evol. 25, 345–353. doi: 10.1016/j.tree.2010.01.007 20188434

[B97] Power J. F. Follett R. F. (1987). Monoculture. Sci. Am. 256, 78–87. doi: 10.1038/scientificamerican0387-78

[B98] Pratap A. Choudhary A. K. Kumar J. (2010). *In vitro* techniques towards genetic enhancement of food legumes–a review. J. Food Legumes 23, 169–185.

[B99] Pratap A. Choudhary A. K. Kumar J. (2018). Potential, constraints and applications of in *vitro* methods in improving grain legumes. Plant Breed. 137, 235–249. doi: 10.1111/pbr.12590

[B100] R Core Team. (2020). R: A language and environment for statistical computing. Vienna, Austria: R Foundation for Statistical Computing. Available at: https://www.R-project.org/.

[B101] Raseduzzaman Md. Jensen E. S. (2017). Does intercropping enhance yield stability in arable crop production? A meta-analysis. Eur. J. Agron. 91, 25–33. doi: 10.1016/j.eja.2017.09.009

[B102] Sampoux J.-P. Giraud H. Litrico I. (2020). Which recurrent selection scheme to improve mixtures of crop species? Theoretical expectations. G3: Genes Genomes Genet. 10, 89–107. doi: 10.1534/g3.119.400809 PMC6945008 31672848

[B103] Sánchez-Bayo F. (2011). “Impacts of agricultural pesticides on terrestrial ecosystems,” in Ecological impacts of toxic chemicals, 63–87.

[B104] Santalla M. Rodino A. P. Casquero P. A. De Ron A. M. (2001). Interactions of bush bean intercropped with field and sweet maize. Eur. J. Agron. 15, 185–196. doi: 10.1016/S1161-0301(01)00104-6

[B105] Seguí-Simarro J. M. Moreno J. B. Guillot Fernández M. Mir R. (2021). “Species with haploid or doubled haploid protocols,” in Doubled haploid technology: volume 1: general topics, alliaceae, cereals (New York, NY, U.S.A: Humana press), 41–103. 10.1007/978-1-0716-1315-3_3 34270025

[B106] Sofo A. Mininni A. N. Ricciuti P. (2020). Soil macrofauna: A key factor for increasing soil fertility and promoting sustainable soil use in fruit orchard agrosystems. Agronomy 10, 456. doi: 10.3390/agronomy10040456

[B107] Sprague G. F. Tatum L. A. (1942). General vs. specific combining ability in single crosses of corn. J. Am. Soc. Agron. 34, 923–932.

[B108] Thierfelder C. Cheesman S. Rusinamhodzi L. (2012). A comparative analysis of conservation agriculture systems: Benefits and challenges of rotations and intercropping in Zimbabwe. Field Crops Res. 137, 237–250. doi: 10.1016/j.fcr.2012.08.017

[B109] Thomas W. T. B. Forster B. P. Gertsson B. (2003). “Doubled haploids in breeding,” in Doubled haploid production in crop plants: a manual (New York, USA: Springer), 337–349.

[B110] Tilman D. (2020). Benefits of intensive agricultural intercropping. Nat. Plants 6, 604–605. doi: 10.1038/s41477-020-0677-4 32483327

[B111] Vanavermaete D. Fostier J. Maenhout S. De Baets B. (2021). Deep scoping: a breeding strategy to preserve, reintroduce and exploit genetic variation. Theor. Appl. Genet. 134, 3845–3861. doi: 10.1007/s00122-021-03932-w 34387711 PMC8580937

[B112] Vandermeer J. H. (1989). The ecology of intercropping (Cambridge: Cambridge University Press). doi: 10.1017/CBO9780511623523

[B113] Weih M. Karley A. J. Newton A. C. Kiær L. P. Scherber C. Rubiales D.. (2021). ‘Grain yield stability of cereal-legume intercrops is greater than sole crops in more productive conditions. Agriculture 11, 255. doi: 10.3390/agriculture11030255

[B114] Weih M.. (2022a). Application of crop growth models to assist breeding for intercropping: Opportunities and challenges. Front. Plant Sci. 13, 720486. doi: 10.3389/fpls.2022.720486 35185972 PMC8854142

[B115] Weih M. Mínguez M. I. Tavoletti S. (2022b). Intercropping systems for sustainable agriculture. Agriculture, 291. MDPI. doi: 10.3390/agriculture12020291

[B116] Weissmann E. A. Yadav R. N. Seth R. Udaya Bhaskar K. (2023). “Principles of variety maintenance for quality seed production,” in Seed science and technology: biology, production, quality (Springer Nature Singapore, Singapore), 153–172.

[B117] Weyen J. (2021). “Applications of doubled haploids in plant breeding and applied research,” in Doubled haploid technology: volume 1: general topics, alliaceae, cereals (New York, NY, U.S.A: Humana press), 23–39. 10.1007/978-1-0716-1315-3_2 34270024

[B118] Willey R. (1979). Intercropping-its importance and its research needs. Part I. Competition and yield advantages. Field Crop Abstr. 32, 1–10.

[B119] Wright A. J. (1985). Selection for improved yield in inter-specific mixtures or intercrops. Theor. Appl. Genet. 69, 399–407. doi: 10.1007/BF00570909 24253909

[B120] Yamamoto T. (1972). Short cut in rice breeding. JARQ J. Agr Res. Quart. 6 (3), 131–135.

[B121] Yan G. Liu H. Wang H. Lu Z. Wang Y. Mullan D.. (2017). Accelerated generation of selfed pure line plants for gene identification and crop breeding. Front. Plant Sci. 8, 1786. doi: 10.3389/fpls.2017.01786 29114254 PMC5660708

[B122] Zustovi R. Landschoot S. Dewitte K. Verlinden G. Dubey R. Maenhout S.. (2024). Intercropping indices evaluation on grain legume-small grain cereals mixture: a critical meta-analysis review. Agron. Sustain. Dev. 44, 5. doi: 10.1007/s13593-023-00934-4

