# Supplementary material for: Harnessing monocrop breeding strategies for intercrops
Source: Front Plant Sci. 2024 May 10;15:1394413. doi: 10.3389/fpls.2024.1394413 (PMC11119317; doi:10.3389/fpls.2024.1394413)
Supplement: Supplementary file 2 [file DataSheet_2.docx]

Supplementary Material

Harnessing sole crop breeding strategies for intercrops

Reena Dubey^1^, Riccardo Zustovi^1^, Sofie Landschoot^1^, Kevin Dewitte^1^, Greet Verlinden^1^, Geert Haesaert^1^, Steven Maenhout^1*^

^1^Department of Plants and Crops, Faculty of Bioscience Engineering, Ghent University, Ghent, Belgium

*** Correspondance:**
steven.maenhout@ugent.be

# Supplementary data and codes: All the codes, data and results related to this paper is available at https://github.com/UGENT-Predictive-breeding/rdubey.

# Supplementary Figures :

## Representation of scenarios used in the paper:


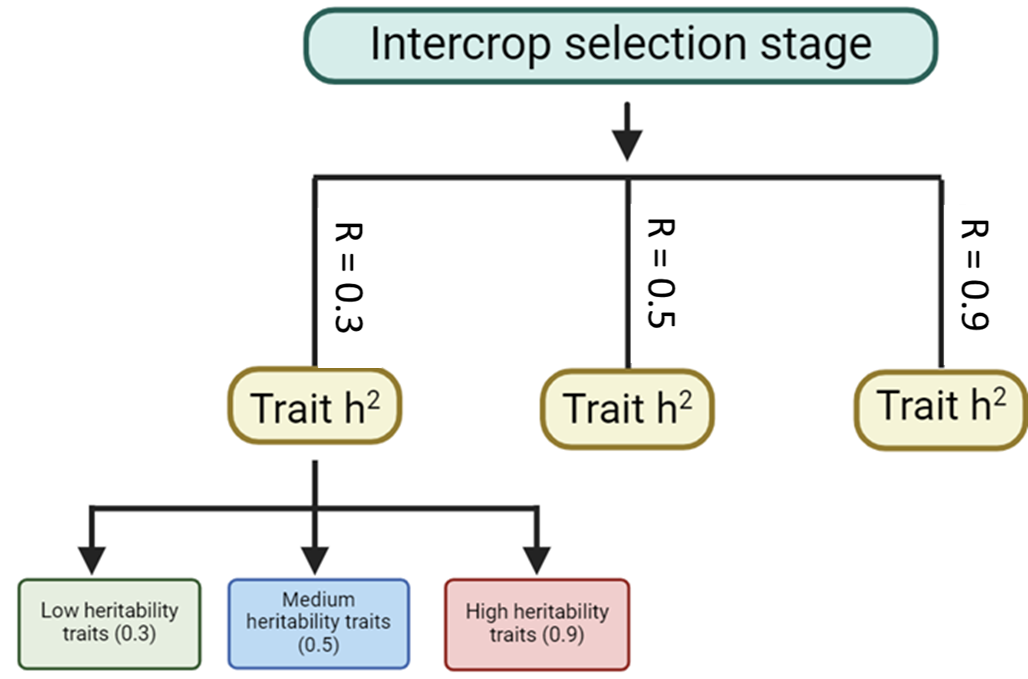


**Supplementary Figure 1.** Each selection stage (Preliminary Yield Trial - PYT, Advanced Yield Trial - AYT, Elite Yield Trial - EYT) mentioned in the paper comprises three correlation scenarios. Within each correlation scenario, there are three levels of heritability: low, medium, and high. This design aims to mimic real traits in crops. In total, there were 54 breeding scenarios encompassing both double haploid (DH) and ear/pod-to-row (EPR) breeding methods.

## Intercrop genetic variance in double haploid breeding method:


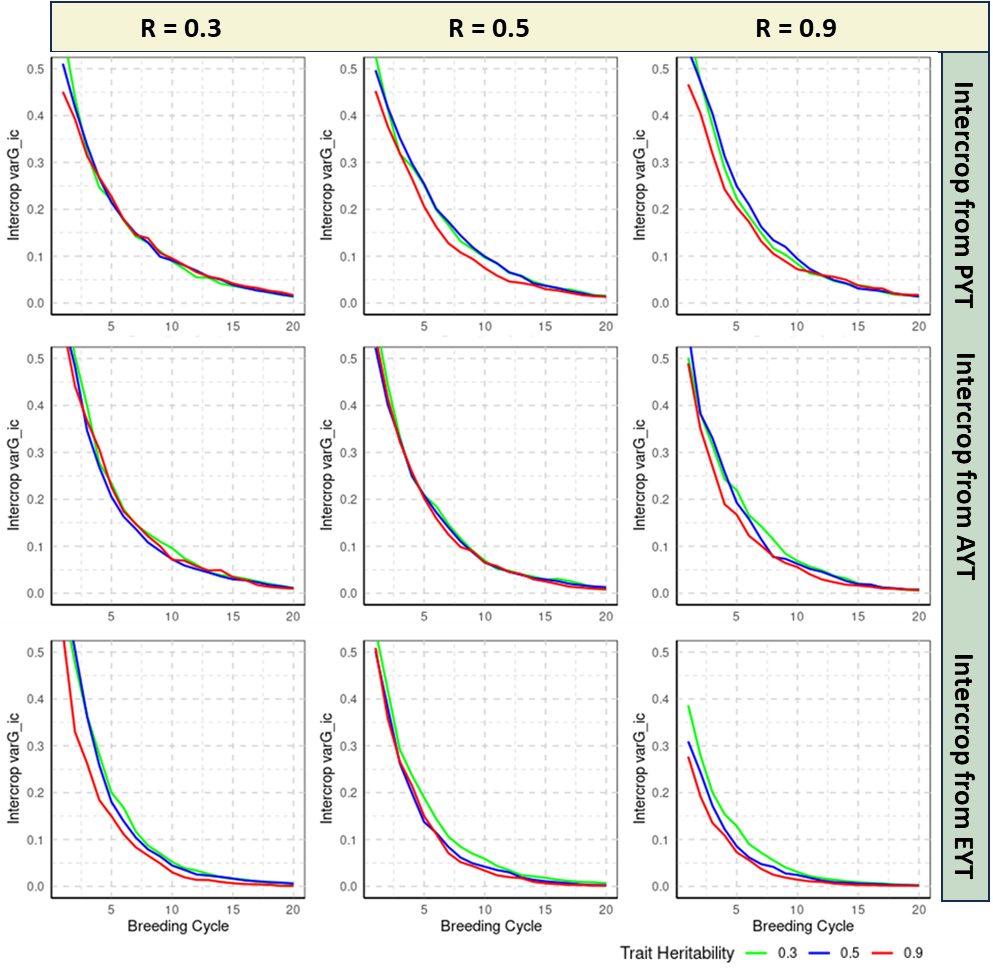


Supplementary Figure 2. Intercrop genetic variance realized by double haploid (DH) breeding scheme as measured by the intercrop (IC) genetic variance(varG) over 20 breeding cycles, shown for three levels of trait heritability (0.3, 0.5, 0.9) and different genetic correlations between MC and IC trait performance ( 0.3, 0.5, 0.9). Phenotypic evaluation of IC performance is either introduced in the Preliminary Yield Trial (PYT), the Advanced Yield Trial (AYT) or the Elite Yield Trial (EYT) selection stages. The realized varG for each breeding method is quantified by plotting the mean IC of the 36 IC combinations at the EYT stage, averaging over 100 independent iterations of the simulation routine.

## Intercrop genetic variance in ear/pod-to-row breeding method:

##
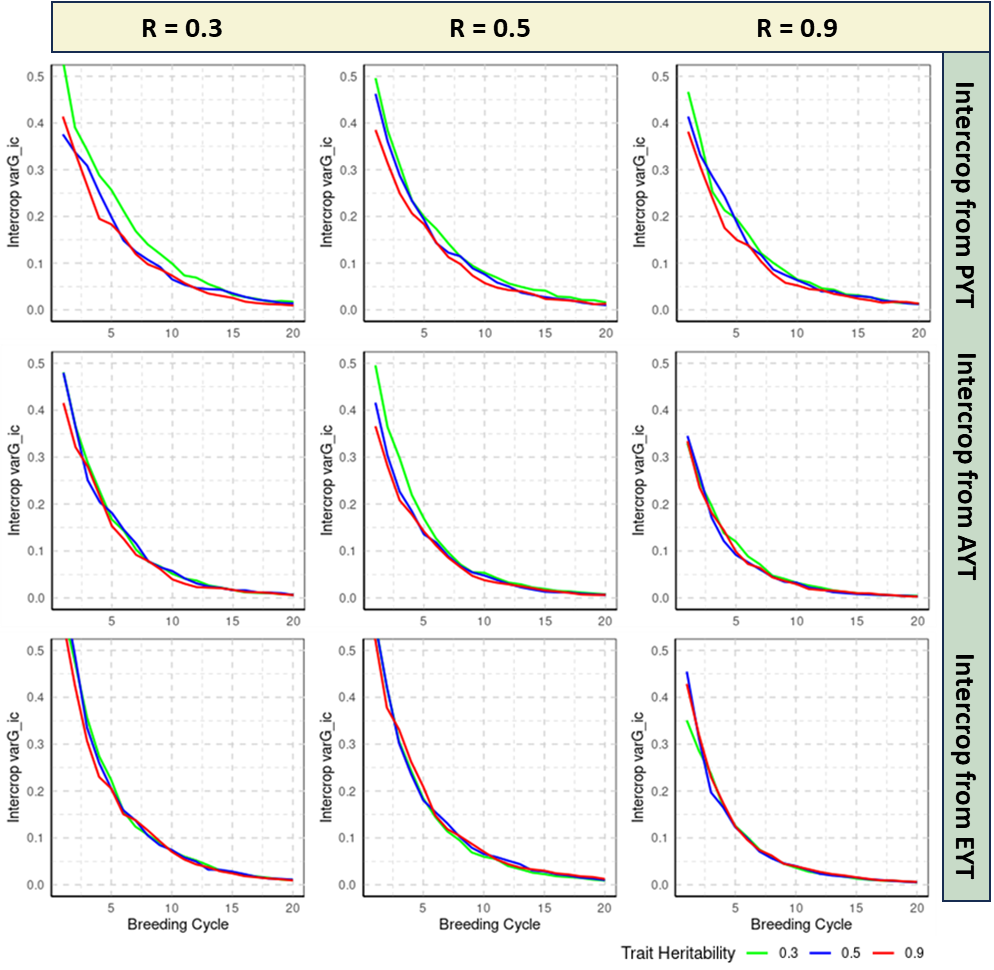


Supplementary Figure 3. Intercrop genetic variance realized by ear/pod-to-row (EPR) breeding scheme as measured by the intercrop (IC) genetic variance(varG) over 20 breeding cycles, shown for three levels of trait heritability (0.3, 0.5, 0.9) and different genetic correlations between MC and IC trait performance ( 0.3, 0.5, 0.9). Phenotypic evaluation of IC performance is either introduced in the Preliminary Yield Trial (PYT), the Advanced Yield Trial (AYT) or the Elite Yield Trial (EYT) selection stages. The realized varG for each breeding method is quantified by plotting the mean IC of the 36 IC combinations at the EYT stage, averaged over 100 independent iterations of the simulation routine.
